# Does three‐dimensional anatomy improve student understanding?

**DOI:** 10.1002/ca.23405

**Published:** 2019-05-31

**Authors:** Charlotte P. R. Triepels, Carlijn F. A. Smeets, Kim J. B. Notten, Roy F. P. M. Kruitwagen, Jurgen J. Futterer, Tineke F. M. Vergeldt, Sander M. J. Van Kuijk

**Affiliations:** ^1^ Department of Obstetrics and Gynaecology Maastricht University Medical Center^+^ Maastricht The Netherlands; ^2^ Department of Obstetrics and Gynaecology Radboud University Medical Centre Nijmegen The Netherlands; ^3^ GROW ‐ School for Oncology and Developmental Biology Maastricht University Medical Centre^+^ Maastricht The Netherlands; ^4^ Department of Radiology and Nuclear Medicine Radboud UMC Nijmegen The Netherlands; ^5^ Department of Clinical Epidemiology and Medical Technology Assessment Maastricht University Medical Centre^+^ Maastricht The Netherlands

**Keywords:** review, three dimensional, traditional methods, anatomy, students

## Abstract

We aim to provide an overview of the various digital three‐dimensional visualizations used for learning anatomy and to assess whether these improve medical students' understanding of anatomy compared to traditional learning methods. Furthermore, we evaluate the attitudes of the users of three‐dimensional visualizations. We included articles that compared advanced newer three‐dimensional anatomy visualization methods (i.e., virtual reality, augmented reality, and computer‐based three‐dimensional visualizations) to traditional methods that have been used for a long time (i.e., cadaver and textbooks) with regard to users' understanding of anatomy. Of the 1,148 articles identified, 21 articles reported data on the effectiveness of using three‐dimensional visualization methods compared to two‐dimensional methods. Twelve articles found that three‐dimensional visualization is a significantly more effective learning method compared to traditional methods, whereas nine articles did not find that three‐dimensional visualization was a significantly more effective method. In general, based on these articles, medical students prefer to use three‐dimensional visualizations to learn anatomy. In most of the articles, using three‐dimensional visualization was shown to be a more effective method to gain anatomical knowledge compared to traditional methods. Besides that, students are motivated and interested in using these new visualization methods for learning anatomical structures. Clin. Anat. 32:25–33, 2019. © 2019 Wiley Periodicals, Inc.

## INTRODUCTION

Medical students often experience difficulties obtaining adequate spatial understanding of three‐dimensional (3D) anatomy from two‐dimensional (2D) images, such as those in anatomy books and on the internet (Battulga et al., 2012; Berney et al., 2015). This may be due to the fact that it is difficult for students in general to mentally rotate static, 2D illustrations (Beermann et al., 2010; Venail et al., 2010). Moreover, due to the complexity of anatomy, medical students and clinicians in training alike experience difficulties in recognizing anatomy in the clinical setting (Smith et al., 2014).

Many different methods are used to learn anatomy, including numerous internet websites dedicated to anatomy images, applications on mobile phones, lectures, oral presentations, 2D pictures (atlases), and cadaveric dissection (Sugand et al., 2010; Estai and Bunt, 2016). Cadaver dissection is often considered to be the gold standard for learning anatomy (Parker, 2002). Currently, more and more 3D visualization methods for teaching anatomy are being developed, at least in part due to the limited availability of cadavers, the high costs associated with obtaining and maintaining them, and the ethical debate surrounding the use of cadavers (McLachlan et al., 2004; Bergman et al., 2011; Ghosh, 2017).

Despite the numerous teaching methods available, many undergraduate and graduate students rate their anatomical knowledge as insufficient (Fitzgerald et al., 2008; Triepels et al., 2018). Many students report being interested in using 3D images to learn anatomy (Bergman et al., 2011; Triepels et al., 2018). A recent study recommended combining 2D and new 3D teaching methods in order to achieve the desired level of anatomical knowledge (Bergman et al., 2011). Yammine and Violato (2015) conducted a meta‐analysis of the effectiveness of using any kind of 3D technology to gain factual and spatial knowledge. They concluded that the use of a 3D tool resulted in higher factual anatomy knowledge and spatial anatomy knowledge compared to traditional methods (Yammine and Violato, 2015). Nevertheless, there is only limited information on the different types of 3D learning methods that are being developed and how effective these different visualization methods are when compared to more traditional anatomy learning methods.

The aim of this review is to provide a comprehensive overview of the various digital 3D visualization methods that have been developed for teaching anatomy and their effectiveness compared to more traditional methods. This review provides the medical educator with a better understanding of digital 3D resources that are available to use. Because of the many differences between the 3D visualization methods that are being developed, we did not aim to pool measures of effectiveness into a single inference. For a meta‐analysis on the topic, see Yammine and Violato (2015).

## MATERIALS AND METHODS

This systematic review is performed in accordance with the guidelines described in the PRISMA statement (Moher et al., 2010).

### Information Sources and Search Strategy

A systematic literature search was performed through computerized databases including Medline, Embase, Cochrane, and Education Resource Information Center. This search was restricted to articles published between 2002 and mid‐2017. The structured PubMed search can be reproduced using the following keywords: (anatomical knowledge or anatomy knowledge or clinical anatomy or anatomy education or anatomical education or “Anatomy/education”[Mesh]) and (3D or 3‐dimensional or three‐dimensional or “Virtual Reality”[MeSH] or digital model or augmented reality or “Imaging, Three‐dimensional”[MeSH]) and (test result or achievement or knowledge or “Knowledge”[MeSH] or cognitive load or skill or effectiveness or opinion or survey or “Surveys and Questionnaires”[MeSH] or attitude or perspective or view or point of view or stance) and (book or textbook or control or traditional or 2D or two‐dimensional or 2‐dimensional or atlas or cadaver or “Cadaver”[MeSH]).

### Selection of Articles

In this study, we selected only original articles that evaluated digital 3D visualization methods for teaching anatomy and compared these to any traditional method based on test results or students' feedback. The 3D visualizations consisted of virtual models (virtual), digital rotatable structures, and augmented reality. The methods were allowed to be based on consecutive 2D images that were placed in three dimensions (such as viewing 3D magnetic resonance imaging [MRI] images based on numerous slices in a single plane). Only articles that had a post‐test design, controlled quasi‐experimental studies, or randomized clinical trials (RCTs) were included. Traditional methods were defined as any method involving anatomy books, training on cadavers, or 2D anatomical pictures. Two authors (C.P.R.T. and C.F.A.S.) independently assessed the title and abstract of each of the articles for eligibility. If at least one of the authors considered one reference eligible, the full text was obtained for complete assessment by a third author. We restricted the search to include articles published from 2002 onward, because the 3D technologies available before that time were considered to be of insufficient relevance because of advancements in digital technology. The selected articles were retrieved for full text review. We excluded articles if they were published in a language other than English, if they dealt with 3D techniques designed to practice surgical procedures, or if they described nondigital 3D visualizations (such as 3D printing and making use of pipe cleaners, glove, or clay models) and if they made use of gross anatomy or dental‐related 3D technology. The references of the included articles were also reviewed for relevant articles that were not found in the search.

The 3D visualization methods were divided into three categories: (1) computer‐based 3D methods, (2) 3D augmented reality methods, and (3) 3D virtual reality methods. According to the Merriam‐Webster dictionary, virtual reality is defined as a technology used to create or access an artificial environment that is experienced through sensory stimuli (such as sights and sounds) provided by a computer and in which one's actions partially determine what happens in the environment. Augmented reality is a technology used to create an enhanced version of reality created by the use of technology to overlay digital information on an image of something being viewed through a device (such as a smartphone camera). The included articles are clustered in the computer‐based 3D methods group when there is no augmented reality nor virtual reality. Computer‐based 3D tools were characterized by electronic and especially computerized technology (Merriam‐Webster's collegiate dictionary, 1999).

### Risk of Bias

We assessed study‐level risk of bias using the Cochrane Risk of Bias Tool (Green et al., 2008). The included articles were categorized as having low risk, high risk, or unclear risk of bias based on items of three domains (i.e., selection bias, detection bias, and attrition bias). Selection bias refers to systematic differences between baseline characteristics of the groups that are compared. Detection bias refers to systematic differences between groups in how outcomes are determined. Attrition bias refers to systematic differences between groups in withdrawals from a study. A judgment of “unclear risk of bias” was made in cases where insufficient information was reported to permit judgment of low or high risk.

## RESULTS

### Study Selection

**Figure 1:**
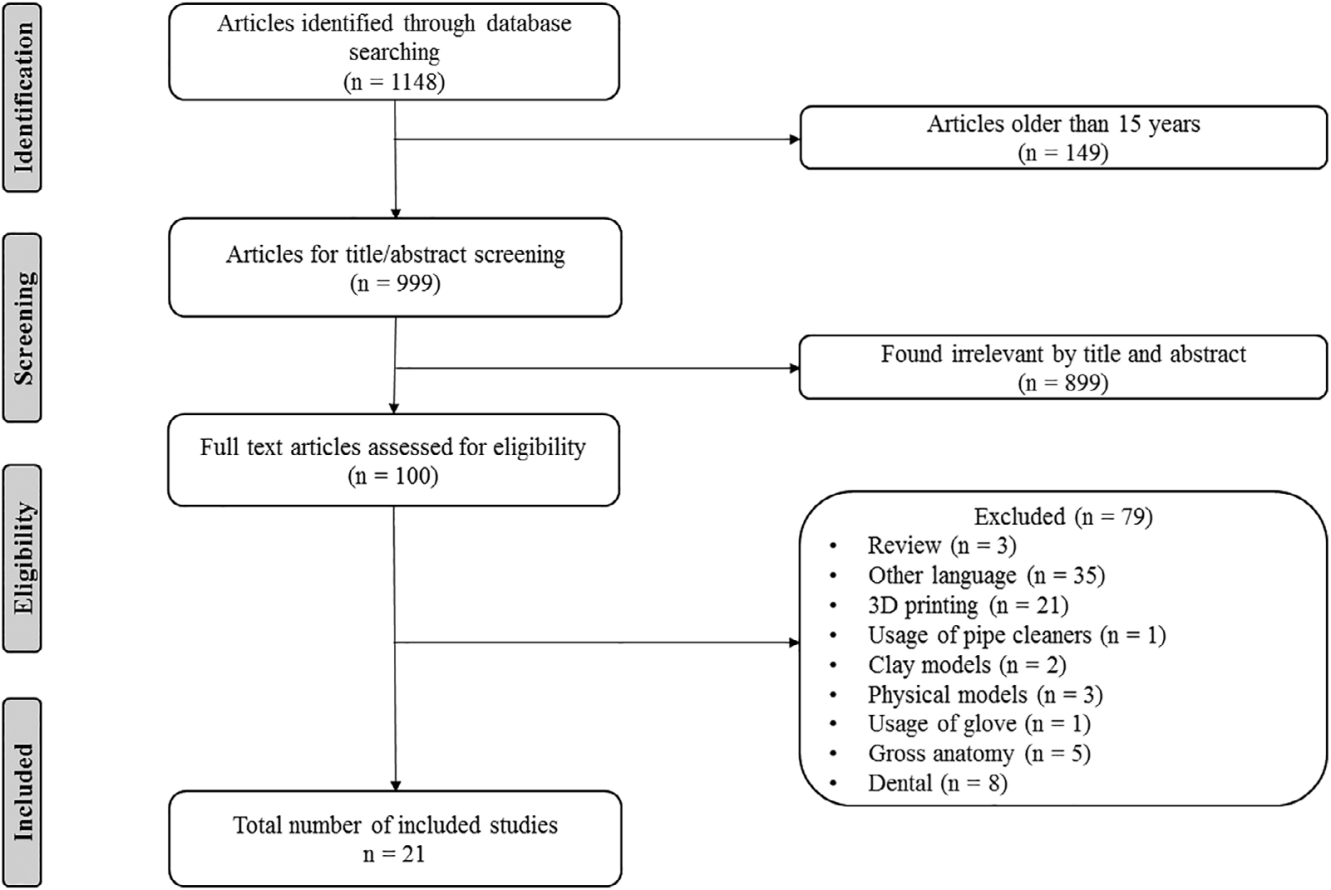
Flowchart of the included articles.

The electronic search strategy identified 1,148 articles that were assessed for eligibility. Twenty‐one articles were selected in which the effectiveness of using 3D visualization methods with using traditional methods was compared. A review of the references of the included articles did not produce any additional articles. The selection process is shown in Figure 1.

The main features of the articles included in this review are show in Table 1. Of the 21 included studies, none were published in the period between 2002 and 2005. Most of the articles were published in the last 10 years. The included studies emerged from different countries, but most often from the United States of America (33%). In regards to the number of participants, most of the articles included fewer than 100 participants except four articles (Venail et al., 2010; Hoyek et al., 2014; Saltarelli et al., 2014; Kockro et al., 2015). An overview of the selected articles including sample size, anatomical structure, study design, type of 3D tool, and study outcomes and differences between groups on the results on the anatomical tests are shown in Table 2. Table 3 shows the study‐level risk of bias. An extensive assessment of the risk of bias can be found in Supporting Information Appendix 1. Of all studies, 12 described using randomization to allocate students to either a 3D visualization method or a traditional method. Naturally, blinding of the participants was not possible in any study because participants would always know to what method they were exposed.

**Table 1:** Main Features of the Manuscripts Included in This Review (*n* = 21)

Features	Number (%)	Included articles
*Publication year*		
2014–2017	8 (38%)	Agbetoba et al. (2017), de Faria et al. (2016), Hoyek et al. (2014), Saltarelli et al. (2014), Viswasom et al. (2017), Kockro et al. (2015), Kucuk et al. (2016), and Peterson and Mlynarczyk (2016)
2010–2013	10 (48%)	Battulga et al. (2012), Brewer et al. (2012), Hassinger et al. (2010), Keedy et al. (2011), Ng et al. (2015), Ruisoto et al. (2012), Tan et al. (2012), Venail et al. (2010), Codd and Choudhury (2011), and Khot et al. (2013)
2006–2009	3 (14%)	Donnelly et al. (2009), Nicholson et al. (2006), and Solyar et al. (2008)
2002–2005	0 (0%)	
*Place of the study*		
United States of America	7 (33%)	Agbetoba et al. (2017), Hassinger et al. (2010), Keedy et al. (2011), Nicholson et al. (2006), Saltarelli et al. (2014), Solyar et al. (2008), and Peterson and Mlynarczyk (2016)
Japan	1 (5%)	Battulga et al. (2012)
Canada	3 (14%)	Brewer et al. (2012), Tan et al. (2012), and Khot et al. (2013)
Brazil	1 (5%)	de Faria et al. (2016)
France	2 (10%)	Hoyek et al. (2014) and Venail et al. (2010)
Asia	1 (5%)	Ng et al. (2015)
Switzerland	1 (5%)	Kockro et al. (2015)
Turkey	1 (5%)	Kucuk et al. (2016)
Spain	1 (5%)	Ruisoto et al. (2012)
India	1 (5%)	Viswasom et al. (2017)
United Kingdom	2 (10%)	Donnelly et al. (2009) and Codd and Choudhury (2011)
*Number of participants*		
0–50	7 (33%)	Agbetoba et al. (2017), Brewer et al. (2012), Hassinger et al. (2010), Keedy et al. (2011), Tan et al. (2012), Codd and Choudhury (2011), and Solyar et al. (2008)
51–100	10 (48%)	Battulga et al. (2012), de Faria et al. (2016), Donnelly et al. (2009), Ng et al. (2015), Nicholson et al. (2006), Ruisoto et al. (2012), Viswasom et al. (2017), Khot et al. (2013), Kucuk et al. (2016), and Peterson and Mlynarczyk (2016)
101–150	0 (0%)	
151–200	2 (10%)	Venail et al. (2010) and Kockro et al. (2015)
>200	2 (10%)	Hoyek et al. (2014) and Saltarelli et al. (2014)
*Three dimensional tool*		
Digital tool	15 (71%)	Viswasom et al. (2017), Venail et al. (2010), Tan et al. (2012), Saltarelli et al. (2014), Ruisoto et al. (2012), Nicholson et al. (2006), Ng et al. (2015), Keedy et al. (2011), Hoyek et al. (2014), Hassinger et al. (2010), Donnelly et al. (2009), de Faria et al. (2016), Brewer et al. (2012), Battulga et al. (2012), and Agbetoba et al. (2017)
Augmented reality tool	2 (10%)	Kucuk et al. (2016) and Peterson and Mlynarczyk (2016)
Virtual reality tool	4 (19%)	Codd and Choudhury (2011), Khot et al. (2013), Kockro et al. (2015), and Solyar et al. (2008)

**Table 2:** Main Features of the Manuscripts Included in This Review (*n* = 21)

	Author and year	Participants (*n*)	Study design	Anatomical structure	3D tool	Study outcomes	Conclusion test
Computer‐based 3D tool	Agbetoba et al. (2017)	45 otorhinolaryngology trainees and 20 medical school students	Multicenter RCT	Neuroanatomy	A preoperative virtual planning software	Subjective opinion (four questionnaires that included a total of 20 items, 10‐point Likert scale)	3D tool is significantly better
	Battulga et al. (2012)	100 participants who had taken anatomy classes and finished cadaver dissection	RCT	Shoulder	3DCG	Subjective opinion (five‐point Likert scale)	3D tool is significantly better
	Brewer et al. (2012)	13 students from a second‐year heath science anatomy course	RCT	Neuroanatomy	A 3D brain model	A post‐test and subjective opinion (four‐point Likert scale)	3D tool is not significantly better
	de Faria et al. (2016)	84 graduate medical students	RCT	Neuroanatomy	An interactive and stereoscopic resource	Written theory test, a lab practicum, and subjective opinion	3D tool is significantly better
	Donnelly et al. (2009)	89 first‐year medical students	RCT	Abdomen	Virtual human dissector	A presession, mid‐session, and postsession test identifying anatomical structures	3D tool is equally effective as a traditional method
	Hassinger et al. (2010)	10 (5 medical students and 5 surgical residents)	Prospective survey based study	Pelvic	Virtual anorectal and pelvic simulator	Subjective opinion according to a combination of five‐point Likert‐scaled items and open‐end questions	3D tool is a useful tool
	Hoyek et al. (2014)	391 students first‐year kinesiology	Quasi experimental design	Upper limb and trunk	3D digital animation	Assessment (20 true/false questions) and subjective opinion (four‐point Likert scale)	Traditional method is significantly better
	Keedy et al. (2011)	46 (19 first‐year and 27 fourth‐year medical students)	RCT	Hepatobiliary	Three dimensional module interface	Nine multiple choice test and subjective opinion (five‐point Likert scale)	3D tool is not significantly better
	Ng et al. (2015)	72 first‐year medical students	RCT	Middle ear	An interactive three‐ dimensional computer model	An anatomy quiz and subjective opinion (four‐Likert scale)	3D tool is significantly better
	Nicholson et al. (2006)	61 first‐year medical students	RCT	Middle and inner ear	Computer‐generated 3D model	A 15‐item quiz	3D tool is significantly better
	Ruisoto et al. (2012)	80 volunteers who are experts, neuropsychologist, neuroanatomist or study psychology/medicine	Multicenter quasi experimental design	Neuroanatomy	3D volumetric visualization	Test (consisting of 36 items) and subjective opinion (five‐point Likert scale)	3D tool is significantly better
	Saltarelli et al. (2014)	214 (mostly [80%] were in their first or second year of university)	Quasi experimental design	Blood vessels in the brain	Anatomy and Physiology revealed (APR) multimedia learning system	Identification and explanation questions	Traditional method is significantly better
	Tan et al. (2012)	40 first‐ and second‐year surgical and anesthesia medical residents	RCT	Larynx	A computer‐generated 3D model	Anatomy test, the modified Vandenberg and Kuse mental rotation test and a subjective opinion (five‐point Likert scale)	3D tool is not significantly better
	Venail et al. (2010)	161 (142 first‐year undergraduate students and 19 otolaryngology fifth‐year residents)	Multicenter quasi‐experimental design	Temporal bone	Computer‐assisted 3D model	Examination and questionnaire (five‐point Likert scale)	3D tool is significantly better
	Viswasom et al. (2017)	94 medical students	Quasi‐experimental design	Osteology	A video demonstrational technique	Examination and subjective opinion	Traditional method is significantly better
Virtual reality	Codd and Choudhury (2011)	39 second years who study the Human Anatomy Research Skills Module	Quasi‐experimental design	Forearm musculoskeletal	3D virtual reality	10 question practical examination and subjective opinion (9 questions)	No significant difference between a 3D tool and traditional methods
	Khot et al. (2013)	60 students with no prior course work in anatomy	RCT	Pelvic	A virtual reality computer‐based module	A Mental Rotations Test and anatomical test (25‐items)	3D tool is equally effective as a traditional method
	Kockro et al. (2015)	169 second‐year medical students	RCT	Third ventricle (neuroanatomy)	3D animated tour with the DextroBeam	10‐question multiple‐choice exam and subjective opinion (4 questions)	3D tool is significantly better
	Solyar et al. (2008)	15 first‐year medical students	RCT	Sinonasal anatomy	Endoscopic surgery simulator	Identification of anatomic structures on a view of nasal cavities and subjective opinion (five‐point Likert scale)	3D tool is significantly better
							
Augmented reality	Kucuk et al. (2016)	70 second‐year undergraduate medical students	RCT	Medulla spinalis ascending and descending pathways	Mobile augmented reality	Test (30 multiple choice questions) and subjective opinion (nine‐point Likert scale)	3D tool is significantly better
	Peterson and Mlynarczyk (2016)	56 (51 graduate and 5 upper level undergraduate students)	Cohort	Not specific	3D augmented material	272 examination questions and a subjective opinion (five‐point Likert scale)	3D tool is significantly better

**Table 3:** Risk of Bias

	Random sequence generation and allocation concealment (selection bias)	Blinding of outcome assessment (detection bias)	Incomplete outcome data (attrition bias)	
Agbetoba et al. (2017)				
Battulga et al. (2012)				
Brewer et al. (2012)				
Codd and Choudhury (2011)				
De Faria et al. (2016)				
Donnelly et al. (2009)				
Hassinger et al. (2010)				
Hoyek et al. (2014)				
Keedy et al. (2011)				Low risk
Khot et al. (2013)				Unclear
Kockro et al. (2015)				High risk
Kucuk et al. (2016)				
Ng et al. (2015)				
Nicholson et al. (2006)				
Peterson and Mlynarczyk (2016)				
Ruisoto et al. (2012)				
Saltarelli et al. (2014)				
Solyar et al. (2008)				
Tan et al. (2012)				
Venail et al. (2010)				
Viswasom et al. (2017)				

### Overview of the Different Three‐Dimensional Methods Used for Learning Anatomy

#### *Computer‐based 3D methods*

Fifteen articles investigated the effectiveness of using a computer‐based 3D computer model to learn anatomy (Table 1). Four RCTs used a neuroanatomical model for the assessment of a computer‐based 3D tool, of which three concluded that the use of a computer‐based 3D methods could improve anatomy teaching. Ruisoto et al. (2012) constructed volumetric images with embedded 3D graphic models from functional positron emission tomography scans. The group exposed to the 3D models showed significantly more correctly identified anatomical structures than a group that studied subcortical structures in 2D cross sections (42.1% compared to 25.4%, scale 0%–100%, *P* < 0.01; Ruisoto et al., 2012). De Faria et al. (2016) assessed an interactive stereoscopic lecture (a computer‐based virtual reality method) that is accessible from personal computers. They concluded that the 3D method resulted in greater improvement in students' anatomical knowledge as the 3D group had significantly higher test scores compared with a group that attended a conventional lecture with 2D images (respectively, 6.45 and 4.36; scale 0–10; *p* < 0.05; de Faria et al., 2016). Agbetoba et al. (2017) evaluated the effectiveness of a tool that permits the learner to draw 3D boxes on relevant anatomical structures and to highlight the frontal sinus outflow pathway. According to their results, most of the students (89.3%) concluded that 3D methods would help them understand spatial orientations (Agbetoba et al., 2017). Additionally, 89.7% of the participants in this study agreed or strongly agreed that they would continue to utilize the 3D software in their clinical practice if the software were available (Agbetoba et al., 2017). Only one study using the neuroanatomical model did not find significantly higher scores for a 3D digital brain model (Brewer et al., 2012). They found that the mean test score after using that model was not significantly better than using 2D images in atlases (23.5% vs. 22.3% on a 0%–100% scale, *P* = 0.95; Brewer et al., 2012).

In addition to neuroanatomy, other anatomical structures have been used to compare the effectiveness of traditional and 3D methods, with conflicting results. Computer‐based 3D models of the ear were proved very promising in two studies (Nicholson et al., 2006; Ng et al., 2015). Ng et al. (2015) used a model constructed with Google Sketchup, which could be used on an iPad. In this study, the 3D group that used anatomical textbooks and a journal article with detailed illustrations of the epitympanum scored significantly higher, achieving a mean score of 65.1% compared to the 2D group's mean score of 32.4% (scale 0%–100%; *P* < 0.001; Ng et al., 2015). Nicholson et al. (2006) used a model constructed from a high‐resolution MRI scan of the middle and inner ear of a human cadaver. The intervention group's mean score on the quiz was significantly higher than the control group's score that used text and 2D images (respectively, 83% and 65%; scale 0%–100%; *P* < 0.001; Nicholson et al., 2006).

Hoyek et al. (2014) used QuickTime Player (Apple) to show 3D structures of the upper limb and trunk that students could pause and rewind. The traditional group was taught with lectures and was presented 2D anatomical pictures. The 2D group outperformed the 3D group on both general knowledge questions (*P* < 0.001) and spatial understanding questions (*P* < 0.001; Hoyek et al., 2014). Donnelly et al. (2009) and Hassinger et al. (2010) both focused on anatomical structures in the abdomen. Donnelly et al. (2009) used “Virtual Human Dissector” software and concluded that the differences in mean scores between intervention and control group were not significant. The study by Hassinger et al. (2010) of relatively low quality (Table 3) used an interactive virtual anorectal and pelvis model. This model was created from magnetic resonance and computed tomography images of a male patient. Structures were colored and labeled with clinically relevant descriptions. Most of the participants (90%) agreed that the simulator is a useful tool for learning anatomy (Hassinger et al., 2010).

In an RCT, Codd and Choudhury (2011) of relatively low quality (Table 3) focused on the anatomy of the liver and biliary system. They used an interactive environment created using Macromedia Flash, in which anatomical structures were labeled (Codd and Choudhury, 2011). The 3D group scored higher than the 2D group (taught using dissection and textbooks) with a mean score of 74% and 64% respectively, although the difference was not statistically significant (*P* = 0.33; Codd and Choudhury, 2011). Saltarelli et al. (2014) explored the effectiveness of a multimedia simulation software that uses high‐resolution illustrations to construct a cadaver and provides animations showing the function of blood vessels in the brain. They concluded that human cadaver dissection offered a significant advantage over the multimedia simulation program (*P* < 0.01). The study by Venail et al. (2010) of relative low quality (Table 3) and a multicenter quasi‐experimental design study aimed to determine if 3D computer software enhanced users' knowledge of the anatomy of the temporal bone. The results of those who took the 3D reconstruction tutorial course (89.92 ± 1.84) were higher than the results of the traditional method group that gets a lecture without a 3D reconstruction (80.91 ± 2.18; scale 0–100; *P* < 0.001; Venail et al., 2010).

Tan et al. (2012) conducted an RCT to investigate the effectiveness of a computer‐generated 3D laryngeal model. This model was constructed using computed tomography and MRI, which were segmented into key structures (Tan et al., 2012). The model was subsequently imported into Microsoft PowerPoint software and enhanced with audio, color, video clips, and clinical vignettes. The traditional method group in this study had a mean score of 15.5 ± 2.3 compared to the 3D group, which had a mean score of 15.7 ± 2.0 (*P* = 0.7222; Tan et al., 2012). Using a quasi‐experimental design study, Viswasom and Jobby (2017) investigated a video demonstration which included 3D views. This study had a relative high risk of bias (Table 3). Test results showed that the mean score of the traditional method group was 5.43, whereas for the 3D group, the mean score was 4.59 (scale unclear, *P* = 0.047; Viswasom and Jobby, 2017). Battulga et al. (2012) employed an RCT that focused on the opinion of medical students regarding the effectiveness of animated and interactive 3D computer graphics (3DCG). They concluded that there was a significant difference in mean scores between the 3DCG and the textbook‐only group (4.26 and 3.85, respectively; five‐point Likert scale; *P* = 0.001; Battulga et al., 2012).

#### *Augmented reality*

Two articles investigated the effectiveness of augmented reality as a learning tool. One study found that although students preferred traditional methods, using augmented reality produced better test results compared to traditional dissection and lecture learning (Peterson and Mlynarczyk, 2016). Additionally, the other study found that test scores of students who used mobile augmented reality were statistically significantly higher than those who used 2D pictures, graphs, and text (*P* < 0.05; Kucuk et al., 2016).

#### *Virtual reality*

Four articles investigated the effectiveness of using virtual reality to learn anatomy. Three out of four articles that investigated the educational effectiveness of virtual reality found that virtual reality methods were more successful than using books alone for studying (Solyar et al., 2008; Codd and Choudhury, 2011; Kockro et al., 2015), although not all statistically significant (*P* > 0.05) (Codd and Choudhury, 2011). Only one study that explored the educational effectiveness of using virtual reality found it offered no advantage over static presentations of 2D anatomical depictions (Khot et al., 2013). The mean test score of the traditional method group (that studied six photographed views of a plastic model) was almost equal to that of the virtual reality group (40% and 41%, respectively, scale 0%–100%; Khot et al., 2013).

### Students' Opinion About Three‐Dimensional Visualization

In 17 of the included articles, a questionnaire was used to measure the user's subjective evaluation of the 3D tool. Most of the participants in the studies reported that the 3D methods were easier and more enjoyable to use. Sometimes, however, due to the complicated anatomical configuration, participants found the 3D methods disorienting and frustrating (Agbetoba et al., 2017). Three articles focused only on the participant's subjective opinion of the tool and did not test the effect of using the tool on the participants' anatomical knowledge (Hassinger et al., 2010; Battulga et al., 2012; Agbetoba et al., 2017); all three of these articles concluded that a 3D tool is useful for learning anatomy.

## DISCUSSION

The relative effectiveness of the use of 3D visualizations was examined in 21 published articles. Twelve articles showed that, according to the users' test results, using a 3D visualization method was significantly more effective than using traditional methods. Although nine articles found no significant difference between the effectiveness of using a 3D visualization method compared to traditional methods, three articles found using the latter to be significantly more effective. Based on three articles which only observed the participants' subjective opinion concluded that 3D methods are useful for learning anatomy. In the computer‐based 3D visualization group, we found 15 articles, whereas in the augmented realty and virtual reality group, we only found two and four articles, respectively. Augmented reality and virtual reality are two relatively new types of 3D visualization techniques and thus do not have a large pool of literature. As a result, it is difficult to provide an overall conclusion of the usefulness of these techniques.

Strengths of this systematic review were the thorough and systematic search and the independent selection of articles and data extraction by multiple authors. Although the aims of the included articles were very similar, there were many differences in the methods used and the primary outcomes that were reported. Because of the variability in the outcomes from the included articles, it was decided to assess the risk of bias of each article.

This systematic review has some limitations. As shown in Table 3, several studies included in this review have a high risk of bias. One frequent reason is the lack of randomization. Selection bias may have occurred in these nonrandomized studies, but it is unclear from the manuscripts to what extent this may have happened. Another limitation is that the included articles are almost all based on different parts of the body. The effectiveness of 3D methods for learning anatomy may be affected by difficulty (Fernandez et al., 2011; Nguyen et al., 2012). For one, learning the anatomy of the medical neurosciences is more difficult than learning musculoskeletal anatomy (Allen et al., 2016). The nervous system is one of the most spatially complex systems of the human body (Brewer et al., 2012) and the shoulder is considered to be one of the most difficult joints for medical students (Battulga et al., 2012). Another source of variation between studies is the fact that participants were included who are in a different phase of their education compared to other studies. For example, some studies' participants are first‐year medical students (Nicholson et al., 2006; Solyar et al., 2008; Donnelly et al., 2009; Ng et al., 2015) and other studies' participants are fifth‐year medical students (Venail et al., 2010). Obviously, the longer medical students study the more basic knowledge of anatomy they have. This makes comparing results between studies complicated. In addition, two of the studies had only a small number of participants, which may have affected their representativeness. For example, Solyar et al. (2008) had only 17 participants and Hassinger et al. (2010) had 10 participants. Other aspects that could have influenced the users scores are the difficulty of the anatomical test and the number of test questions. Some of the included articles did not assess the difficulty of the anatomical structures and/or the difficulty of the questions on the anatomical test.

In conclusion, the included studies demonstrate that computer‐based, virtual reality and augmented reality 3D learning methods in general are more effective means of learning anatomy, based on users' test scores, compared to traditional methods. However, the techniques varied greatly, and more research into augmented and virtual reality should be performed as the number of studies on those techniques was low. In most studies, participants state that they prefer to learn anatomical structures using a 3D tool.

## Supporting information

Appendix 1: Risk of bias

## References

[ca23405-bib-0001] Agbetoba A , Luong A , Siow JK , Senior B , Callejas C , Szczygielski K , Citardi MJ . 2017 Educational utility of advanced three‐dimensional virtual imaging in evaluating the anatomical configuration of the frontal recess. Int Forum Allergy Rhinol 7:143–148. 27754596 10.1002/alr.21864 PMC5299043

[ca23405-bib-0002] Allen LK , Eagleson R , de Ribaupierre S . 2016 Evaluation of an online three‐dimensional interactive resource for undergraduate neuroanatomy education. Anat Sci Educ 9:431–439. 26990135 10.1002/ase.1604

[ca23405-bib-0003] Battulga B , Konishi T , Tamura Y , Moriguchi H . 2012 The effectiveness of an interactive 3‐dimensional computer graphics model for medical education. Interact J Med Res 1:e2. 23611759 10.2196/ijmr.2172 PMC3626131

[ca23405-bib-0004] Beermann J , Tetzlaff R , Bruckner T , Schoebinger M , Muller‐Stich BP , Gutt CN , Meinzer HP , Kadmon M , Fischer L . 2010 Three‐dimensional visualisation improves understanding of surgical liver anatomy. Med Educ 44:936–940. 20716104 10.1111/j.1365-2923.2010.03742.x

[ca23405-bib-0005] Bergman EM , van der Vleuten CP , Scherpbier AJ . 2011 Why don't they know enough about anatomy? A narrative review. Med Teach 33:403–409. 21355704 10.3109/0142159X.2010.536276

[ca23405-bib-0006] Berney S , Betrancourt M , Molinari G , Hoyek N . 2015 How spatial abilities and dynamic visualizations interplay when learning functional anatomy with 3D anatomical models. Anat Sci Educ 8:452–462. 25689057 10.1002/ase.1524

[ca23405-bib-0007] Brewer DN , Wilson TD , Eagleson R , de Ribaupierre S . 2012 Evaluation of neuroanatomical training using a 3D visual reality model. Stud Health Technol Inform 173:85–91. 22356963

[ca23405-bib-0008] Codd AM , Choudhury B . 2011 Virtual reality anatomy: Is it comparable with traditional methods in the teaching of human forearm musculoskeletal anatomy? Anat Sci Educ 4:119–125. 21480538 10.1002/ase.214

[ca23405-bib-0009] de Faria JW , Teixeira MJ , de Moura Sousa Junior L , Otoch JP , Figueiredo EG . 2016 Virtual and stereoscopic anatomy: When virtual reality meets medical education. J Neurosurg 125:1105–1111. 26871375 10.3171/2015.8.JNS141563

[ca23405-bib-0010] Donnelly L , Patten D , White P , Finn G . 2009 Virtual human dissector as a learning tool for studying cross‐sectional anatomy. Med Teach 31:553–555. 19288305 10.1080/01421590802512953

[ca23405-bib-0011] Estai M , Bunt S . 2016 Best teaching practices in anatomy education: A critical review. Ann Anat 208:151–157. 26996541 10.1016/j.aanat.2016.02.010

[ca23405-bib-0012] Fernandez R , Dror IE , Smith C . 2011 Spatial abilities of expert clinical anatomists: Comparison of abilities between novices, intermediates, and experts in anatomy. Anat Sci Educ 4:1–8. 21265030 10.1002/ase.196

[ca23405-bib-0013] Fitzgerald JE , White MJ , Tang SW , Maxwell‐Armstrong CA , James DK . 2008 Are we teaching sufficient anatomy at medical school? The opinions of newly qualified doctors. Clin Anat 21:718–724. 18773486 10.1002/ca.20662

[ca23405-bib-0014] Ghosh SK . 2017 Cadaveric dissection as an educational tool for anatomical sciences in the 21st century. Anat Sci Educ 10:286–299. 27574911 10.1002/ase.1649

[ca23405-bib-0038] Green S , Higgins JPT. 2008 Green JPHaS: Cochrane Handbook for Systematic Reviews of Interventions. England: John Wiley & Sons Ltd.

[ca23405-bib-0015] Hassinger JP , Dozois EJ , Holubar SD , Camp JC , Farley DR , Fidler JL , Pawlina W , Robb RA , Larson DW . 2010 Virtual pelvic anatomy simulator: A pilot study of usability and perceived effectiveness. J Surg Res 161:23–27. 19959192 10.1016/j.jss.2009.06.016

[ca23405-bib-0016] Hoyek N , Collet C , Di Rienzo F , De Almeida M , Guillot A . 2014 Effectiveness of three‐dimensional digital animation in teaching human anatomy in an authentic classroom context. Anat Sci Educ 7:430–437. 24678034 10.1002/ase.1446

[ca23405-bib-0039] Keedy AW , Durack JC , Sandhu P , Chen EM , O'Sullivan PS , Breiman RS . 2011 Comparison of traditional methods with 3D computer models in the instruction of hepatobiliary anatomy. Anat Sci Educ 4:84–91. 21412990 10.1002/ase.212

[ca23405-bib-0017] Khot Z , Quinlan K , Norman GR , Wainman B . 2013 The relative effectiveness of computer‐based and traditional resources for education in anatomy. Anat Sci Educ 6:211–215. 23509000 10.1002/ase.1355

[ca23405-bib-0018] Kockro RA , Amaxopoulou C , Killeen T , Wagner W , Reisch R , Schwandt E , Gutenberg A , Giese A , Stofft E , Stadie AT . 2015 Stereoscopic neuroanatomy lectures using a three‐dimensional virtual reality environment. Ann Anat 201:91–98. 26245861 10.1016/j.aanat.2015.05.006

[ca23405-bib-0019] Kucuk S , Kapakin S , Goktas Y . 2016 Learning anatomy via mobile augmented reality: Effects on achievement and cognitive load. Anat Sci Educ 9:411–421. 26950521 10.1002/ase.1603

[ca23405-bib-0020] McLachlan JC , Bligh J , Bradley P , Searle J . 2004 Teaching anatomy without cadavers. Med Educ 38:418–424. 15025643 10.1046/j.1365-2923.2004.01795.x

[ca23405-bib-0021] Moher D , Liberati A , Tetzlaff J , Altman DG , Group P . 2010 Preferred reporting items for systematic reviews and meta‐analyses: The PRISMA statement. Int J Surg 8:336–341. 20171303 10.1016/j.ijsu.2010.02.007

[ca23405-bib-0022] Ng CL , Liu X , Chee SC , Ngo RY . 2015 An innovative 3‐dimensional model of the epitympanum for teaching of middle ear anatomy. Otolaryngol Head Neck Surg 153:832–837. 25994233 10.1177/0194599815584600

[ca23405-bib-0023] Nguyen N , Nelson AJ , Wilson TD . 2012 Computer visualizations: Factors that influence spatial anatomy comprehension. Anat Sci Educ 5:98–108. 22232125 10.1002/ase.1258

[ca23405-bib-0024] Nicholson DT , Chalk C , Funnell WR , Daniel SJ . 2006 Can virtual reality improve anatomy education? A randomised controlled study of a computer‐generated three‐dimensional anatomical ear model. Med Educ 40:1081–1087. 17054617 10.1111/j.1365-2929.2006.02611.x

[ca23405-bib-0025] Parker LM . 2002 What's wrong with the dead body? Use of the human cadaver in medical education. Med J Aust 176:74–76. 11936290 10.5694/j.1326-5377.2002.tb04290.x

[ca23405-bib-0026] Peterson DC , Mlynarczyk GS . 2016 Analysis of traditional versus three‐dimensional augmented curriculum on anatomical learning outcome measures. Anat Sci Educ 9:529–536. 27078503 10.1002/ase.1612

[ca23405-bib-0027] Ruisoto P , Juanes JA , Contador I , Mayoral P , Prats‐Galino A . 2012 Experimental evidence for improved neuroimaging interpretation using three‐dimensional graphic models. Anat Sci Educ 5:132–137. 22434672 10.1002/ase.1275

[ca23405-bib-0028] Saltarelli AJ , Roseth CJ , Saltarelli WA . 2014 Human cadavers Vs. multimedia simulation: A study of student learning in anatomy. Anat Sci Educ 7:331–339. 24415563 10.1002/ase.1429

[ca23405-bib-0029] Smith CF , Martinez‐Alvarez C , McHanwell S . 2014 The context of learning anatomy: Does it make a difference? J Anat 224:270–278. 23930933 10.1111/joa.12089 PMC3931538

[ca23405-bib-0030] Solyar A , Cuellar H , Sadoughi B , Olson TR , Fried MP . 2008 Endoscopic sinus surgery simulator as a teaching tool for anatomy education. Am J Surg 196:120–124. 18374890 10.1016/j.amjsurg.2007.06.026

[ca23405-bib-0031] Sugand K , Abrahams P , Khurana A . 2010 The anatomy of anatomy: A review for its modernization. Anat Sci Educ 3:83–93. 20205265 10.1002/ase.139

[ca23405-bib-0032] Tan S , Hu A , Wilson T , Ladak H , Haase P , Fung K . 2012 Role of a computer‐generated three‐dimensional laryngeal model in anatomy teaching for advanced learners. J Laryngol Otol 126:395–401. 22075619 10.1017/S0022215111002830

[ca23405-bib-0033] Triepels CPR , Koppes DM , Van Kuijk SMJ , Popeijus HE , Lamers WH , van Gorp T , Futterer JJ , Kruitwagen R , Notten KJB . 2018 Medical students' perspective on training in anatomy. Ann Anat 217:60–65. 29501634 10.1016/j.aanat.2018.01.006

[ca23405-bib-0034] Venail F , Deveze A , Lallemant B , Guevara N , Mondain M . 2010 Enhancement of temporal bone anatomy learning with computer 3D rendered imaging software. Med Teach 32:e282–e288. 20653370 10.3109/0142159X.2010.490280

[ca23405-bib-0035] Viswasom AA , Jobby A . 2017 Effectiveness of video demonstration over conventional methods in teaching osteology in anatomy. J Clin Diagn Res 11:JC09–JC11. 10.7860/JCDR/2017/24029.9429 PMC5376855 28384890

[ca23405-bib-0037] Yammine K , Violato C . 2015 A meta‐analysis of the educational effectiveness of three‐dimensional visualization technologies in teaching anatomy. Anat Sci Educ 8:525–538. 25557582 10.1002/ase.1510

